# Inflammatory myofibroblastic tumor: a rare laryngeal case

**DOI:** 10.1590/S1808-86942010000200020

**Published:** 2015-10-19

**Authors:** Flavio Carlos, Mirelle Limp Boa Vida, Bruno Bernardo Duarte, Flavio Akira Sakae, Silvio Antonio Monteiro Marone

**Affiliations:** Medical resident at the ORL Unit, Pontificial Catholic University (PUC), Campinas; Medical resident at the ORL Unit, PUC, Campinas; Medical resident at the ORL Unit, PUC, Campinas; Assisting physician, ORL Unit, PUC, Campinas. Doctoral student in ORL, Medical School of the Sao Paulo University; Full professor of the ORL Unit, PUC, Campinas

**Keywords:** larynx, tumor

## INTRODUCTION

The inflammatory myofibroblastic tumor (IMT) is more commonly found in the lungs.^1^^,^^2^ The paranasal sinuses and the orbit are the most affected areas in the head and neck; the laryngeal presentation is extremely rare, and only about 15 cases have been described in the literature. We report the clinical findings of a case of laryngeal IMT that was seen at the otorhinolaryngology unit of a university hospital.

## CASE REPORT

**Figure 1:**
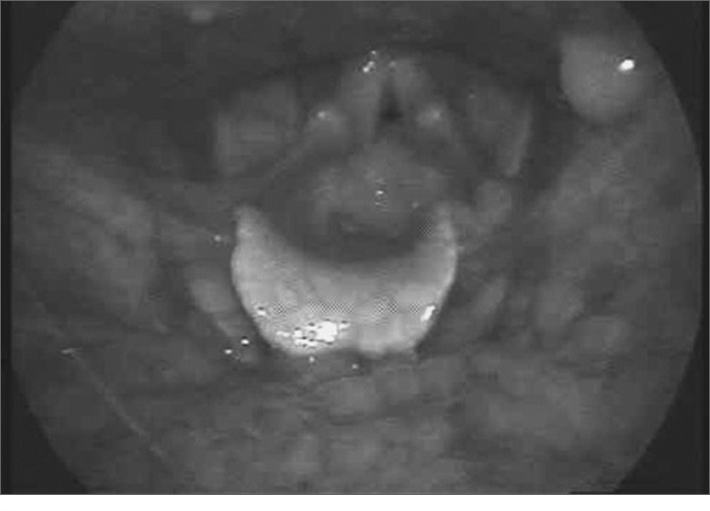
Laryngeal endoscopy showed a granulomatous lesion within nearly the entire glottis.

A retired female patient aged 71 years, from the city of Campinas, presented to the outpatient unit complaining of persistent dysphonia for the past two years. There was no dyspnea or dysphagia. The patient had a history of voice abuse, gastroesophageal reflux, and smoking for 30 years that she had interrupted 12 years ago. She reported not consuming alcoholic beverages and undergoing radiotherapy for thyroid cancer 22 years ago. Laryngeal endoscopy revealed a granulomatous lesion on the lower two thirds of the left vocal fold, which took up most of the glottis (Fig. 1); the vocal folds were mobile. The possible diagnoses were vocal polyp, papilloma, granuloma, and squamous cell carcinoma; laryngeal microsurgery was chosen as the preferred approach. The lesion was hard, there was no cleavage plane with the border of the vocal fold, and the lesion was located within Reinke's space. The lesion was removed fully. Pathology revealed an IMT, and immunohistochemical analysis was positive for vimentin and smooth muscle actin; it was negative for pancytokeratins and the epithelial membrane antigen. Cervical tomography and chest X-rays were within normal limits, and laryngeal endoscopy one year after surgery showed no recurrence. Dysphonia regressed significantly.

## DISCUSSION

Laryngeal IMT appears to progress slowly; its outcome is uncertain, given the rarity of this lesion. Wenig et al.^3^ studied 8 laryngeal IMT cases among which recurrence was found in only one case. These authors described laryngeal IMT as a nonmetastasizing lesion that may rarely recur locally or as multifocal lesions.

The etiology is uncertain; factors such as trauma,^1^ the Epstein-Barr virus,^4^ and chromosome translocation (2p23), and the gene ALK^2^ have been suggested as likely causes. Exposure to radiotherapy may be related to the onset of this tumor in our case.

Guilemany et al.^2^ reported 11 cases in adults aged from 19 to 74 years (8 male and 3 female). Four cases were reported in children^4^^,^^5^ aged from 5 to 11 years (3 male and 1 female).

The clinical presentation ranges from dysphonia and stridor to respiratory failure. Guilemany et al.^2^ described the first case in the larynx with systemic manifestations - fever, anemia, thrombocytosis and altered erythrocyte sediment. The patient in another reported case1 had ossifying myositis.

The glottis is most commonly affected in the larynx, as a polypoid pedunculated mass with an irregular surface. The differential diagnosis includes vocal polyps, laryngeal papillomas, granulomas, and malignancies.

Histologically, IMT consists of fusiform cells with fibroblasts and myofibroblasts, and a lymphocytic and plasmacytic infiltrate. It may be differentiated from fusiform cell carcinoma, fibrous histiocitoma, fibrosarcoma, nodular fasciitis, myogenic tumors, myofibroblastoma, fibromatosis, and congenital infantile fibrosarcoma.

The diagnosis is made with immunohistochemical methods, which are vimentin positive in 89–99% of cases, smooth muscle specific in 89% of cases, and smooth muscle actin positive in 92% of cases.^2^

The best surgical approach appears to consist of excision biopsy. Guilemany et al.,^2^ in a review of the literature, found one case in which cordectomy was done followed by radiotherapy and, eventually, total laryngectomy. Radiotherapy may be used for the treatment of persistent lesions.5

Excision biopsy of the lesion yielded excellent functional results in our patient, since voice was preserved and no recurrences were seen one year later.

## FINAL COMMENTS

A rare case of an inflammatory myofibroblastic tumor in the larynx was described; the results were satisfactory following excision biopsy of the lesion.
